# #Metoo or #Hertoo? A Moderated Mediation Model of Gender Differences in Perceptions of Sexual Harassment

**DOI:** 10.1007/s10508-022-02344-1

**Published:** 2022-07-20

**Authors:** Heather M. Clarke

**Affiliations:** Austin E. Cofrin School of Business, University of Wisconsin-Green Bay, WH 460, 2420 Nicolet Drive, Green Bay, WI 54311-7001 USA

**Keywords:** Sociosexual behaviors, Sexual harassment, Gender differences, Sexual orientation, Discrimination, Moderated mediation

## Abstract

Sexual harassment continues to pervade workplaces due, at least in part, to gender differences in the perception of sociosexual behaviors. Some scholars have argued that such differences are minimal and inconsistent. This study examined and demonstrated several reasons why this conclusion is fallacious. Approximately equal numbers of gay men (*n* = 191), heterosexual men (*n* = 193), lesbians (*n* = 190), and heterosexual women (*n* = 196) reported their perceptions of scenarios describing an interaction between a target and their manager. The target was either a fictional female, a fictional male, or the participant. As predicted, only heterosexual men’s perceptions of sociosexual behaviors varied by the target of the behaviors. Heterosexual men viewed the behaviors as harassment only when the target was female. Further, women and gay men, but not heterosexual men, viewed the sociosexual behaviors as discrimination. The results also supported a moderated mediation model where, following exposure to sociosexual behaviors, the effect of participant group on perceived sexual harassment was mediated by fear and perceived discrimination and moderated by target. This study contributes to research on workplace sexual harassment by explaining alleged inconsistent results of studies of gender differences in perceptions of sexual harassment and by proposing and testing a novel process following exposure to sociosexual behaviors in the workplace.

## Introduction

Sexual harassment continues to plague American workplaces despite being deemed illegal sex discrimination by the United States Supreme Court thirty-five years ago (*Meritor Savings Bank v. Vinson*, 1986). The extent of its prevalence is unknown because only a minority of incidents are ever reported (e.g., Society for Human Resource Management, 2018). However, the #metoo movement founded by Tarana Burke and a myriad of media accounts of high-profile perpetrators (see, e.g., Carlsen et al., 2018), have brought to light that workplace sexual harassment remains a serious problem. One reason for this may be that gender differences in the psychological experience of sociosexual behaviors prevent many men from taking the perspective of sexual harassment targets (Clarke, 2020).

Several gender differences related to sexual harassment find support in the literature. For example, female targets experience worse outcomes than male targets (e.g., Chan et al., 2008), women are targeted more frequently (e.g., Rospenda et al., 2009), and men are more likely to be harassers (e.g., Berdahl & Raver, 2011). Another significant gender difference, germane to this research, is that women are more likely to perceive sociosexual behaviors to be sexual harassment (e.g., Berdahl et al., 1996). Some researchers, however, have argued that there is little difference between the way women and men experience sexual harassment (e.g., Blumenthal, 1998). Indeed, studies on perceptions of sexual harassment support both the presence (e.g., Dillon et al., 2015) and absence (e.g., Castillo et al., 2011) of gender differences.

The purpose of the study reported herein was to clarify the inconsistent findings on gender differences in perceptions of sociosexual behavior in the workplace and to examine whether men, particularly heterosexual men, experience sociosexual behaviors differently than women. This study examined four factors potentially determinative of gender differences and makes several contributions to scholarly knowledge of workplace sexual harassment. First, I examined the impact of perspective-taking on perceptions of sexual harassment to explain the inconsistent findings in extant research. Vignette studies have employed “imagine-other” perspective-taking or “imagine-self” perspective-taking. This is the first published study on workplace sexual harassment to incorporate and compare both. Second, I investigated whether gender differences in perceptions of sexual harassment are explained not only by the extent to which sociosexual behaviors are experienced as threatening, but also by the extent to which they are experienced as discriminatory. Third, rejecting the heteronormative assumption underlying research in this area, I examined whether gay men’s perceptions of sexual harassment differ from those of heterosexual men. Finally, I tested a novel psychological process that occurs following exposure to sociosexual behaviors in the workplace.

What follows are, in turn, an examination of gender differences in perceptions of sociosexual behavior in the workplace, an explication of the method, delineation of the results, and a discussion of the theoretical and practical implications of the findings.

### Gender Differences in Perceptions of Sociosexual Behavior

Sociosexual behavior is “any non-work-related behavior having a sexual component” (Gutek et al., 1990, p. 560). Sociosexual behavior includes a wide range of behaviors such as comments, looks, and gestures, whether complimentary or insulting, as well as touching and requests for dates. These behaviors may be perceived as benign or even pleasurable, such as flirting (Gutek et al., 1990); however, they can also be perceived as threatening, degrading, or humiliating. That is, they can be perceived as sexual harassment. The U.S. Equal Employment Opportunity Commission defines sexual harassment as “unwelcome sexual advances, requests for sexual favors, and other verbal or physical harassment of a sexual nature” (n.d.).

Meta-analyses and reviews of the literature on gender differences in perceptions of sexual harassment have questioned the existence and/or magnitude of such differences due to inconsistent findings in the literature (Blumenthal, 1998; Gutek, 1995; Rotundo et al., 2001). Blumenthal (1998), for example, concluded that gender differences were negligible. However, these meta-analyses and reviews have not distinguished among survey studies of lived experiences, labeling studies, and vignette studies that employ perspective-taking. This is problematic because study design determines the construct that is being measured.

Some survey studies ask participants to report their own experiences of sexual harassment. Although unacknowledged, the published studies measuring lived experiences included in Blumenthal’s (1998) meta-analysis reported significant gender differences. Female participants were harassed at higher rates, were more likely to experience sociosexual behaviors as harassment, and were more likely to report experiencing negative consequences as a result (Komaromy et al., 1993; Konrad & Gutek, 1986; Morrow et al., 1994).

Labeling studies present behaviors, either as a list or as brief scenarios, and ask participants whether they are sexual harassment. These studies do not measure perceptions of sexual harassment. They merely solicit an objective definition or label and often produce no gender differences (e.g., Bursik & Gefter, 2011). Knowledge of which behaviors are deemed to be sexual harassment may be acquired from many sources, including sexual harassment training or media reports. Labeling a behavior sexual harassment is not akin to experiencing it as such. Early sexual harassment research demonstrated that individuals could experience sociosexual behaviors as harassing without labeling them to be harassment (Magley et al., 1999; Munson et al., 2001). I posit that the converse is also true; individuals can label behaviors sexual harassment without experiencing them as harassing. It is therefore intuitive that there would be no gender differences in labeling behaviors to be sexual harassment, and these studies cannot be tendered as evidence of a lack of gender differences.

A third type of study, the vignette study, is commonplace in workplace sexual harassment research because true experiments are limited for ethical reasons. The use of perspective-taking in vignette studies influences what is being measured and whether gender differences will be found.

### The Role of Perspective-Taking

Two types of perspective-taking can be found in sexual harassment research. The first is “imagine-other” perspective-taking (Batson et al., 1997; Chambers & Davis, 2012; Davis et al., 2004). Imagine-other perspective-taking, also known as mind-reading (Ames et al., 2012), involves inferring what the fictional target in the scenario thinks and feels. This form of perspective-taking is employed when participants are instructed to make inferences about how a fictional target in a scenario would perceive or experience the situation (e.g., Castillo et al., 2011). According to similarity-contingency theory (Ames et al., 2012), individuals are more likely to project their own beliefs and attitudes on a target if they perceive themselves to be similar to the target, whereas individuals are more likely to rely on stereotypes and outside information when they perceive themselves to be dissimilar to the target.

When making inferences about a fictional target of sociosexual behaviors, female participants are more likely than male participants to perceive similarity to the target because they are more likely to have experienced similar behaviors themselves. Per similarity-contingency theory, then, female participants are more likely to project their own perceptions onto the target and male participants are more likely to rely upon stereotypes and outside information. Female participants are more likely to perceive sociosexual behaviors as harassment and project that perception onto the fictional target. Male participants, relying on stereotypes and objective knowledge of what is harassment, will also infer that the fictional target perceives the behaviors to be harassment. Although arrived at through difference psychological processes, male and female participants in an imagine-other perspective-taking study would likely respond similarly.

The second type of perspective-taking is called “imagine-self” (Batson et al., 1997; Chambers & Davis, 2012; Davis et al., 2004). Imagine-self perspective-taking requires an individual to imagine what they would think and feel if the described situation happened to them. Like survey studies of lived experiences, these vignette studies capture participants’ own perceptions and produce gender differences (e.g., Dillon et al., 2015). None of the vignette studies included in Blumenthal’s (1998) meta-analysis that produced either null or mixed findings employed imagine-self perspective-taking (Baker et al., 1990; Bursik, 1992; Dietz-Uhler & Murrell, 1992; Egbert et al., 1992; Hartnett et al., 1989; Jones & Remland, 1992; McKinney, 1992; Moore et al., 1994; Oswald & Caudill, 1991; Summers, 1992; Terpstra & Baker, 1987; Thomann & Wiener, 1987). The imagine-self perspective-taking studies produced gender differences (Garlick, 1994; Lester et al., 1986; Thacker & Gohmann, 1993; Valentine-French & Radtke, 1989). When imagine-self perspective-taking is employed, females will be more likely than males to perceive sociosexual behaviors to be sexual harassment.

### Appraisal of Sociosexual Behaviors

Sexual harassment has been identified as a workplace stressor that leads to a wide array of negative psychological, physical, and job-related outcomes (e.g., Fitzgerald et al., 1997; Hershcovis & Barling, 2010), either directly (e.g., Kath et al., 2009), or indirectly through emotions such as fear (e.g., Barling et al., 2001). However, early sexual harassment research demonstrated that individuals could experience sociosexual behaviors as harassment without labeling them as such (Magley et al., 1999; Munson et al., 2001). It is therefore likely that an emotional response to the behaviors precedes, rather than succeeds, the perception of the behaviors as sexual harassment. That is, a target experiences fear in response to sociosexual behaviors which leads to perceiving the behaviors to be sexual harassment, and female targets are more likely than male targets to experience fear in response to sociosexual behaviors.

Sociosexual behaviors are not always of a nature that would evoke fear but may be viewed as unequal treatment, or discrimination, which then leads to the perception of the behavior as sexual harassment. Workplace sexual harassment is illegal because it is a form of sex discrimination, yet scant organizational psychology research has conceptualized sexual harassment as discrimination (e.g., Shaffer et al., 2000). Some psychology research has explicitly distinguished sexual harassment from discrimination (e.g., Chan et al., 2008). It appears that no published studies have examined whether harassment is perceived to be employment discrimination since Murrell et al. (1995). Yet sexual harassment is an exercise of power and used to maintain social dominance over the target (Berdahl, 2007; Clarke, 2020). Clarke et al. (2016) posited that due to women’s historical and persisting inequality in the workplace, being the target of sociosexual behaviors at work is a reminder of that inequality. Thus, not only are women more likely than men to experience fear in response to sociosexual behaviors, but they are also more likely to experience them as workplace discrimination. Per the discussion of perspective-taking above, these gender differences would only manifest in “imagine-self” perspective-taking studies and not in “imagine-other” studies.

### Gay Men

Research on gender differences in perceptions of sexual harassment typically does not include sexual orientation as a variable nor report the sexual orientation of the study participants. However, it may be that gay men have different perceptions of sociosexual behaviors in the workplace than heterosexual men because gay men are targets of sexual harassment and sexual violence more often than heterosexual men (Chen et al., 2020). Further, gay men experience prejudice, discrimination, and sexual orientation harassment in the workplace (e.g., Horvath & Ryan, 2003; Ryan & Wessel, 2012). Thus, gay men may be more likely than heterosexual men to experience fear in response to sociosexual behaviors and/or to perceive them as discriminatory, resulting in perceptions of sexual harassment. Again, this group difference would only be elicited by “imagine-self” perspective-taking.

### Hypothesis

Based on the forgoing, I predict a three-way interaction of sociosexual behaviors, participant, and target such that, in the sociosexual behaviors condition, but not the control condition:

**Hypothesis 1**: Heterosexual males, but not females or gay males, will report higher (a) perceived discrimination, (b) fear, and (c) perceived sexual harassment when the target is fictional (imagine-other perspective-taking) than when they are the target (imagine-self perspective-taking).

**Hypothesis 2**: Heterosexual males will report lower (a) perceived discrimination, (b) fear, and (c) perceived sexual harassment than females and gay males when they are the target (imagine-self perspective-taking), but not when the target is fictional (imagine-other perspective-taking).

**Fig. 1:**
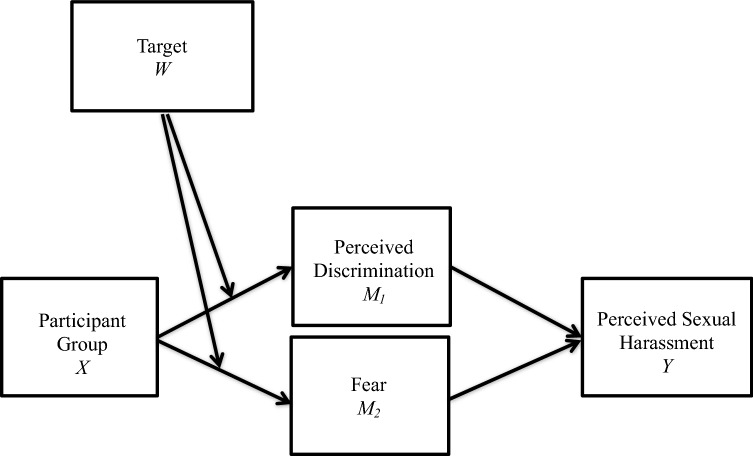
Conceptual model predicting moderated parallel mediation

Further, when subjected to sociosexual behaviors in the workplace, females and gay males will be more likely than heterosexual males to experience fear and perceptions of discrimination, which will in turn predict perceptions of sexual harassment. In addition, these two indirect effects will be moderated by the target of the sociosexual behaviors, such that participant group will predict fear and perceived discrimination ratings when the target of the behaviors is the participant, but not when the target is fictional. Therefore, when exposed to sociosexual behaviors in the workplace:

**Hypothesis 3**: Perceptions of sexual harassment will be predicted by a moderated mediation model such that participant group (heterosexual male, gay male, heterosexual female, or lesbian) will predict perceived sexual harassment through the mediators of fear and perceived discrimination when the target is the participant (imagine-self perspective-taking), but not when the target is fictional (imagine-other perspective-taking) (Figure 1).

## Method

I carried out a vignette study that followed a 2 × 3 × 4 design: sociosexual behaviors condition (control or sociosexual behaviors) x target (fictional female, fictional male, or participant) x participant group (lesbian, heterosexual female, gay male, or heterosexual male). The scenarios described an interaction between the target and their manager at work. I employed a between-subject experimental design to avoid demand and carryover effects. (To avoid confusing the participant as target condition with the participant group conditions, I hereinafter refer to the participant as target condition as the “perspective-taking” condition.)

### Participants

I recruited approximately equal numbers of heterosexual, gay, and lesbian employed adults in the USA through Amazon Mechanical Turk (MTurk), a crowdsourcing internet marketplace. (Although I did not predict differences between lesbian and heterosexual female participants, differences may exist because there is evidence that females who are sexual minorities experience more harassment than heterosexual females (SteelFisher et al., 2019). Thus, I recruited them as separate groups.) MTurk provides a forum where Requesters post Human Intelligence Tasks or HITS, which Workers complete for payment. MTurk provides a mechanism through which Requesters can reject a Worker’s work if they failed to complete the work as instructed. To bolster data quality, I recruited only Workers that had at least a 95% approval rating (i.e., less than 5% of the work they had previously completed on MTurk had been rejected by other Requesters). The use of MTurk permitted the recruitment of employed adults in the USA, the target population. The ability to screen MTurk Workers for sexual orientation also enabled the attainment of equal numbers of gay male, lesbian, and heterosexual male and female participants.

The total number of participants recruited was 876. After removing incomplete questionnaires, and data due to failed attention and manipulation checks (target gender), the total number of participants was 770 (lesbian = 190, heterosexual female = 196, gay male = 191, heterosexual male = 193). The ages of the participants ranged from 18 to 71 years with a mean of 32.9 years. The distribution of participant race was: White = 71.4%, Black or African-American = 12.1%, Hispanic or Latino = 7.5%, Asian = 7.3%, and Other = 1.7%.

### Procedure

MTurk Workers who met my requirements (resident in the USA and at least a 95% approval rating) were able to view the HITS I posted on MTurk to recruit study participants. To recruit approximately equal numbers of male and female and heterosexual and gay/lesbian participants, I posted separate HITS for each of these four groups as well as for each of the two sociosexual behavior conditions and the three target conditions. This resulted in a total of 24 HITS in order to randomly assign each of the four groups of participants to each of the six conditions. The numbers of participants per condition are reported in Table 1.

**Table 1:** Participants by condition

Participant	Target					
	Control			Sociosexual behaviors		
	Female	Male	Perspective-taking	Female	Male	Perspective-taking
Lesbian	30	37	29	30	34	30
Heterosexual Female	33	36	30	33	34	30
Gay Male	31	34	30	29	35	32
Heterosexual Male	32	36	31	32	32	30

MTurk Workers followed a link to the online study materials on Qualtrics, indicated their consent to participate, and read one of six scenarios. The scenarios were modified versions of the scenarios used by Clarke et al. (2016) and are reproduced in Appendix A. Both the control scenario and the sociosexual behavior scenario were identical across the three targets with only the identity of the target and the instructions changing. In the perspective-taking condition, the target was referred to as “you” and participants were given perspective-taking instructions that directed them to imagine that the scenario was happening to them (i.e., imagine-self perspective-taking instructions; Chambers & Davis, 2012; Davis et al., 2004). In the female and male target conditions, the target was called Mary or Mark, respectively, and participants were simply directed to read the scenario.

The scenario described an interaction between the target and their manager. With a view to bolstering the realism of the scenario, the gender of the manager was not disclosed so that participants could impute gender based on their own conceptualization or experience of sexual harassment and/or experience with managers. In the control conditions, the scenario described a collegial interaction with the manager. In the sociosexual behaviors condition, the scenario described an interaction in which the manager exhibited sociosexual behaviors (touching, leering, and sexual comments) toward the target. The sociosexual behaviors were forms of unwanted sexual attention like those appearing in the Sexual Experiences Questionnaire (SEQ; Fitzgerald & Shullman, 1985; Fitzgerald et al., 1988) and used in prior studies (e.g., Berdahl et al., 1996; Clarke et al., 2016).

After reading the scenario, participants then completed a questionnaire containing the items listed below, along with attention questions to ensure data quality. After completing the questionnaire, they were directed to a debriefing webpage.

### Measures

#### Perceived Sexual Harassment

Participants reported the extent to which they agreed that they/Mary/Mark (depending on the condition) were/was sexually harassed in the scenario on a five-point scale (1 = strongly disagree, 5 = strongly agree).

#### Fear

The extent to which the target would feel afraid following the incident in the scenario was measured with four items reported on a seven-point scale from 1 = strongly disagree to 7 = strongly agree. Sample items are: “I/Mary/Mark would fear that what I experienced in the scenario would happen again” and “What happened in the scenario would make me/Mary/Mark feel afraid.” Cronbach’s alpha = 0.96.

#### Perceived Discrimination

Participants reported the extent to which they agreed that they/Mary/Mark were/was discriminated against in the scenario on a five-point scale (1 = strongly disagree, 5 = strongly agree).

#### Past Sexual Harassment

Participants indicated whether they had experienced workplace sexual harassment (yes/no). The percentage of each participant group that reported experiencing workplace sexual harassment were as follows: lesbian = 35.8%, heterosexual female = 38.3%, gay male = 35.4%, and heterosexual male = 15.2%.

#### Target Gender Manipulation Check

I included items to verify the successful manipulation of gender in the female and male target conditions and removed the data of any participants that responded incorrectly.

#### Demographics

Participants reported their gender and sexual orientation. Given the four groups the study was designed to compare, only those reporting to be male or female and heterosexual or gay/lesbian were included in the study. Participants also reported their age and race (Asian, Black or African-American, Hispanic or Latino, White, or Other).

## Results

Means on the dependent variables by condition are reported in Table 2. Correlations, overall means, and standard deviations for study variables are given in Table 3.

**Table 2:** Means and SDs by condition

Dependent variable	Participant	Target					
		Control			Sociosexual behaviors		
		Female	Male	Perspective-taking	Female	Male	Perspective-taking
Perceived Sexual Harassment^a^	Lesbian	1.53 (1.17)	2.16 (1.42)	1.41 (.98)	4.33 (1.09)	4.32 (.81)	4.24 (.87)
	Heterosexual Female	1.36 (1.08)	2.47 (1.73)	1.23 (.77)	4.70 (.53)	4.32 (.88)	4.67 (.48)
	Gay Male	1.77 (1.28)	2.82 (1.57)	2.20 (1.50)	4.31 (.97)	4.11 (.63)	3.75 (1.32)
	Heterosexual Male	1.16 (.57)	1.64 (1.25)	1.39 (.96)	4.62 (.75)	3.75 (1.16)	3.70 (1.39)
Perceived Discrimination^a^	Lesbian	1.47 (.97)	2.27 (1.43)	1.38 (.86)	3.80 (1.30)	3.94 (1.15)	3.79 (1.32)
	Heterosexual Female	1.30 (.92)	2.53 (1.54)	1.27 (.64)	3.94 (1.03)	3.65 (1.28)	3.27 (1.39)
	Gay Male	1.97 (1.38)	2.76 (1.54)	1.87 (1.22)	3.79 (1.78)	3.86 (1.12)	3.44 (1.39)
	Heterosexual Male	1.25 (.62)	1.72 (1.26)	1.55 (1.03)	3.94 (1.24)	3.34 (1.18)	2.27 (1.34)
Fear^b^	Lesbian	2.06 (1.27)	2.98 (1.76)	2.12 (1.312)	5.31 (1.35)	5.47 (1.19)	5.29 (1.22)
	Heterosexual Female	2.20 (1.51)	3.82 (1.82)	2.75 (1.57)	5.91 (.84)	5.22 (1.12)	5.58 (1.12)
	Gay Male	2.98 (1.75)	3.74 (1.81)	2.82 (1.64)	5.71 (.77)	5.24 (.84)	4.84 (1.31)
	Heterosexual Male	2.06 (1.01)	2.83 (1.77)	2.56 (1.43)	5.82 (.99)	4.62 (1.15)	4.25 (1.66)

^a^Absolute range: 1–5

^b^Absolute range: 1–7

**Table 3:** Means, SDs, and zero-order correlations

	M	SD	1	2	3	4	5	6	7
1. Participant gender^a^	–	–	1						
2. Participant sexual orientation^b^	–	–	.01	1					
3. Perceived discrimination^c^	2.69	1.57	.03	.13^**^	1				
4. Fear^d^	4.01	1.93	.03	.03	.72^**^	1			
5. Perceived sexual harassment^c^	3.00	1.70	.04	.07	.79^**^	.81^**^	1		
6. Past sexual harassment^e^	–	–	.13^**^	.11^**^	.19^**^	.15^**^	.17^**^	1	
7. Age^f^	32.90	9.68	.02	− .26^**^	− .10^**^	− 06	− 06	.01	1

^a^Male = 0, female = 1

^b^Heterosexual = 0, lesbian/gay = 1

^c^Absolute range: 1–5

^d^Absolute range: 1–7

^e^No = 0, yes = 1

^f^Absolute range: 18–71

^*^p < .05, two-tailed

^**^p < .01, two-tailed

### Hypotheses 1 and 2

Because the three outcome variables were correlated, I tested Hypotheses 1 and 2 with a three-way (sociosexual behavior condition x target x participant group) MANCOVA. I included past sexual harassment and age as covariates as they were correlated with one or more dependent variables. The result indicated that the three-way interaction was significant: *F*(18, 737) = 2.02, *p* = 0.007, Wilk's Λ = 0.952.

There was a significant main effect of sociosexual behaviors on perceived discrimination: *F*(1, 729) = 435.25, *p* < 0.001, η_p_^2^ = 0.374, fear: *F*(1, 729) = 644.27, *p* < 0.001, η_p_^2^ = 0.469, and perceived sexual harassment: *F*(1, 729) = 978.83, *p* < 0.001, η_p_^2^ = 0.573. Perusal of the means, provided in Table 2, indicated that the sociosexual behaviors condition was rated higher than the control condition on all three outcome variables. As predicted in Hypotheses 1 and 2, the main effects were qualified by the three-way interaction of sociosexual behavior x target x participant group.

#### Perceived Discrimination

The result of the test of the three-way interaction on perceived discrimination was: *F*(6, 729) = 2.86, *p* = 0.009, η_p_^2^ = 0.023. To test Hypotheses 1a and 2a, I probed this interaction with a two-way (target x participant group) ANCOVA within each sociosexual behavior condition. The result for the control condition was not significant (*p* > 0.025). The result for the sociosexual behaviors condition was significant (*F*(6, 357) = 2.62, *p* = 0.017, η_p_^2^ = 0.042). Then, to test Hypothesis 1a, I conducted four one-way ANCOVAs, comparing perceived discrimination ratings across targets within each participant group. As predicted, the results for lesbians, heterosexual females, and gay males were not significant (*p* > 0.0125) and the result for heterosexual males was significant (*F*(2, 86) = 13.28, *p* < 0.001, η_p_^2^ = 0.236). Examination of the means in Table 2 indicates that heterosexual males rated the female target condition (M = 3.94, SD = 1.24) higher than both the perspective-taking (M = 2.27, SD = 1.34) and male target (M = 3.34, SD = 1.18) conditions. Thus, Hypothesis 1a was largely supported. Perceived discrimination ratings varied by target only for heterosexual males and both the female and male target conditions were rated higher than the perspective-taking condition; however, the female target condition was also rated higher than the male target condition.

I then tested Hypothesis 2a with three one-way ANCOVAs, comparing perceived discrimination ratings across participants within each target group. The results for the female target and male target conditions were not significant (*p* > 0.017), but the result for the perspective-taking condition was significant (*F*(3, 112) = 5.96, *p* = 0.001, η_p_^2^ = 0.138). Perusal of Table 2 indicates that, in the perspective-taking condition, the mean for heterosexual males (M = 2.27, SD = 1.34) was lower than the means for gay males (M = 3.44, SD = 1.39), lesbians (M = 3.79, SD = 1.32), and heterosexual females (M = 3.27, SD = 1.39). The mean for heterosexual males indicated that, on average, they were the only participants that did not perceive the behaviors to be discrimination, supporting Hypothesis 2a.

#### Fear

Per Hypotheses 1b and 2b, the three-way interaction of sociosexual behavior condition x target x participant was significant for fear: *F*(6, 729) = 2.86, *p* = 0.009, η_p_^2^ = 0.023. To probe this interaction, I first conducted a two-way (target x participant) ANCOVA within each sociosexual behavior condition. For the control condition, the result was not significant (*p* > 0.025). The result for the sociosexual behavior condition was significant (*F*(6, 357) = 3.79, *p* = 0.001, η_p_^2^ = 0.060). To test Hypothesis 1b, I conducted four one-way ANCOVAs, comparing fear ratings across targets within each participant group. Consistent with the hypothesis, the results for lesbians, heterosexual females, and gay males were not significant (*p* > 0.0125) and the result for heterosexual male participants was significant (*F*(2, 86) = 14.28, *p* < 0.001, η_p_^2^ = 0.249). Examination of the means in Table 2 indicates that heterosexual males rated the female target condition (*M* = 5.82, SD = 0.99) higher than the perspective-taking (*M* = 4.25, SD = 1.66) and male target (*M* = 4.62, SD = 1.15) conditions. Hypothesis 1b was partially supported and the results mirror those for perceived discrimination. Fear ratings varied significantly by target only for heterosexual male participants, who rated the female and male target conditions higher than the perspective-taking condition. However, they also rated the female target condition higher than the male target condition.

I tested Hypothesis 2b with three one-way ANCOVAs, comparing fear ratings across participants within each target group. As predicted, the results for the female target and male target were not significant (*p* > 0.017), but the result for the perspective-taking condition was significant (*F*(3, 112) = 5.63, *p* = 0.001, η_p_^2^ = 0.131). Perusal of Table 2 indicates that in the perspective-taking condition, the mean for the heterosexual male participants (*M* = 4.25, SD = 1.66) is lower than the means for gay male (*M* = 4.84, SD = 1.12), lesbian (*M* = 5.29, SD = 1.22), and heterosexual female (*M* = 5.58, SD = 1.39) participants, supporting Hypothesis 2b. Heterosexual males reported experiencing less fear than the other three groups.

#### Perceived Sexual Harassment

The three-way interaction for perceived sexual harassment was significant: *F*(6, 729) = 2.45, *p* = 0.023, η_p_^2^ = 0.020. To probe this interaction, I first conducted a two-way (target x participant) ANCOVA within each sociosexual behavior condition. For the control condition, the result was not significant (*p* > 0.025). The result for the sociosexual behavior condition was significant (*F*(6, 357) = 2.41, *p* = 0.027, η_p_^2^ = 0.039). To test Hypothesis 1c, I conducted four one-way ANCOVAs, comparing perceived sexual harassment ratings across targets within each participant group. Consistent with the hypothesis, the results for lesbians, heterosexual females, and gay males were not significant (*p* > 0.0125) and the result for heterosexual males was significant (*F*(2, 86) = 7.10, *p* < 0.001, η_p_^2^ = 0.142). Examination of the means in Table 2 indicates that heterosexual males rated the female target condition (*M* = 4.62, SD = 0.75) higher than the perspective-taking (*M* = 3.70, SD = 1.39) and the male target (*M* = 3.75, SD = 1.16) conditions. Hypothesis 1c was therefore partially supported. Heterosexual males’ ratings were higher for the female target condition than for the male target and perspective-taking conditions, but the ratings of the other three groups did not differ across targets.

I tested Hypothesis 2c with three one-way ANCOVAs, comparing perceived sexual harassment ratings across participants within each target group. The results for the female and male targets were not significant (*p* > 0.017), but the result for the perspective-taking condition was significant (*F*(3, 112) = 4.41, *p* = 0.006, η_p_^2^ = 0.106). Perusal of Table 2 indicates that in the perspective-taking condition, the means for the heterosexual males (*M* = 3.70, SD = 1.39) and gay males (*M* = 3.75, SD = 1.32) were lower than those for lesbians (*M* = 4.24, SD = 0.87) and heterosexual females (*M* = 4.67, SD = 0.48), partially supporting Hypothesis 2c. Heterosexual males and gay males reported lower perceived sexual harassment than lesbians and heterosexual females, but only in the perspective-taking condition.

### Hypothesis 3

Hypothesis 3 predicted a moderated mediation model where, in the sociosexual behaviors condition, participant group would predict perceived sexual harassment ratings both directly and indirectly through perceived discrimination and fear (Fig. 1). Further, the effect of participant group on perceived discrimination and fear would be moderated by target condition. I tested this moderated mediation model using the PROCESS macro in SPSS (version 3.0; Hayes, 2018). I created a custom model with participant condition (heterosexual male, gay male, heterosexual female, or lesbian) as the independent variable, target condition (perspective-taking, female target, or male target) as the moderator, perceived discrimination and fear as the parallel mediators, and perceived sexual harassment as the dependent variable. Past sexual harassment and age were included as covariates.

Because the independent variable (participant group) is a multicategorical variable with four categories, PROCESS created three indicator (or dummy) variables that collectively represent the variable of participant group. Since I predicted that the heterosexual male group would differ from the three other groups of participants, I set heterosexual males as the reference group (Hayes, 2018). As explained in Table 4, the three indictor variables (X1…X3) represent the difference between each of the other three groups and the heterosexual male group. Similarly, the moderator variable (target) is represented by two indicator variables, each contrasting the perspective-taking condition (reference group) with the female (W1) and male (W2) target conditions.

**Table 4:** Interpretations of indicator variables

Variable role	Variable	Indicator	Interpretation
Predictor (*X*)	Participant group	X1	Difference between group 2 (gay males) and group 1 (heterosexual males)
		X2	Difference between group 3 (heterosexual females) and group 1 (heterosexual males)
		X3	Difference between group 4 (lesbians) and group 1 (heterosexual males)
Moderator (*W*)	Target	W1	Difference between target 2 (female target) and target 1 (perspective-taking)
		W2	Difference between target 3 (male target) and target 1 (perspective-taking)

The results of the conditional process analysis are reported in Table 5 through 10. Direct and indirect effects model coefficients for the moderated parallel mediation model are reported in Table 5. I adopted a conservative *p*-value of 0.01 as recommended by Hayes and Preacher (2014) when using multicategorical variables in conditional process analysis.

**Table 5:** Direct and indirect effects model coefficients for the moderated parallel mediation model

Antecedent	Consequent														
	Discrimination (*M*_1_)					Fear (*M*_2_)					Sexual harassment (*Y*)				
		Coeff.	*SE*	LLCI	ULCI		Coeff.	*SE*	LLCI	ULCI		Coeff.	*SE*	LLCI	ULCI
Constant		2.338	.313	1.527	3.149		4.313	.287	3.570	5.055		1.635	.249	.990	2.281
X1	*a*_11_	1.100	.317	.279	1.922	*a*_12_	.561	.290	− 191	1.313	${c}_{1}^{^{\prime}}$	− 220	.124	− 540	.100
X2	*a*_21_	.952	.319	.127	1.777	*a*_22_	1.288	.292	.533	2.043	${c}_{2}^{^{\prime}}$	.157	.123	− 161	.474
X3	*a*_31_	1.442	.323	.606	2.278	*a*_32_	1.122	.296	.356	1.887	${c}_{3}^{^{\prime}}$	− 034	.125	− 358	.291
W1	*b*_11_	1.713	.313	.903	2.522	*b*_12_	1.610	.286	.868	2.351	–	–	–	–	–
W2	*b*_21_	.995	.321	.164	1.826	*b*_22_	.284	.294	− 477	1.044	–	–	–	–	–
Int.1	*a*_11_*b*_11_	− 1.411	.450	− 2.576	− 246	*a*_12_*b*_12_	− 818	.412	− 1.885	.248	–	–	–	–	–
Int.2	*a*_11_*b*_21_	− 645	.446	− 1.800	.509	*a*_12_*b*_22_	.008	.408	− 1.049	1.065	–	–	–	–	–
Int.3	*a*_21_*b*_11_	− 959	.440	− 2.097	.180	*a*_22_*b*_12_	− 1.208	.403	− 2.250	− 165	–	–	–	–	–
Int.4	*a*_21_*b*_21_	− 662	.445	− 1.814	.491	*a*_22_*b*_22_	.691	.407	− 1.747	.364	–	–	–	–	–
Int.5	*a*_3_ *b*_11_	− 1.613	.454	− 2.789	− 438	*a*_32_*b*_12_	− 1.651	.412	− 2.726	− 575	–	–	–	–	–
Int.6	*a*_31_*b*_21_	− 819	.449	− 1.981	.343	*a*_32_*b*_22_	− 244	.411	− 1.307	.820	–	–	–	–	–
Discrim. (*M*_*1*_)	–	–	–	–	–	–	–	–	–	–	*d*_1_	.204	.036	.112	.296
Fear (*M*_*2*_)	–	–	–	–	–	–	–	–	–	–	*d*_2_	.291	.038	.192	.389
Past SH (*cov*_*1*_)	*cov*_1_	.383	.144	.010	.755	*cov*_1_	.362	.132	.021	.702	*cov*_1_	.131	.094	− 112	.373
Age (*cov*_*2*_)	*cov*_2_	− 005	.007	− 023	.013	*cov*_2_	− 004	.006	− 021	.012	*cov*_2_	.010	.005	− 002	.021
	*R*^2^ = .145 *F*(13,357) = 4.65, *p* < .001					*R*^2^ = .179 *F*(13,357) = 5.97, *p* < .001					*R*^2^ = .334 *F*(7,363) = 25.97, *p* < .001				

Discrim./discrimination = perceived discrimination; sexual harassment = perceived sexual harassment; past SH = past experience with sexual harassment; age = participant age; X1…X3 = independent indicator variables; *Y* = dependent variable; W1 and W2 = moderator indicator variables; Int.1…Int.6 = interaction of independent indicator variables with moderator indicator variables; *M*_1_ = mediator 1; *M*_2_ = mediator 2; *cov*_*1*_ and *cov*_*2*_ = covariate; LLCI = lower level of 99% confidence interval; ULCI = upper level of 99% confidence interval

#### Relative Indirect Effects

The indices of moderated mediation for perceived discrimination, provided in Table 6, represent the differences between the conditional indirect effects at the two values of the moderator (i.e., W1 and W2). All confidence intervals are 99% percentile bootstrap confidence intervals based on 10,000 bootstrap samples. As indicated in the table, two indices had confidence intervals that did not include zero: the index for *X*1*W*1 = 0.289 (bootstrap SE = 0.154, LLCI =  − 0.675, ULCI = -0.020) and the index for *X*3*W*1 =  − 0.330 (bootstrap SE = 0.125, LLCI =  − 0.714, ULCI =  − 0.063). Per Hayes (2018), with a multicategorical *X*, “*X*’s indirect effect is moderated if at least one of the bootstrap confidence intervals for the indices of moderated mediation for the relative indirect effects does not contain zero.” Therefore, the indirect effect of participant group on perceived sexual harassment through perceived discrimination was moderated by target.

**Table 6:** Indices of moderated mediation (difference between conditional indirect effects) for the mediator perceived discrimination

Participant group indicator variable	Target indicator variable	Index	Boot *SE*	Boot LLCI	Boot ULCI
X1	W1	− 289	.125	675	− 020
	W2	− 132	.102	− 452	.108
X2	W1	− 196	.106	− 517	.039
	W2	− 135	.101	− 437	.098
X3	W1	− 330	.125	− 714	− 063
	W2	− 168	.099	− 452	.070

Probing the moderation of mediation requires examination of the relative indirect effects at the three values of the moderator, provided in Table 7. The relative indirect effects for all three participant group comparisons were significant only in the perspective-taking condition: *X*1 = 0.225 (bootstrap SE = 0.097, LLCI = 0.023, ULCI = 0.533), *X*2 = 0.195 (bootstrap SE = 0.088, LLCI = 0.001, ULCI = 0.455), *X*3 = 0.295 (bootstrap SE = 0.098, LLCI = 0.086, ULCI = 0.579). This means that the relative indirect effects of the comparisons of heterosexual males with gay males (*X*1), heterosexual males with heterosexual females (*X*2), and heterosexual males with lesbians (*X*3), on perceived sexual harassment through perceived discrimination, were significant in the perspective-taking condition, but not in the female or male target conditions, supporting Hypothesis 3.

**Table 7:** Relative conditional indirect effects of participant group on perceived sexual harassment through perceived discrimination

Participant group indicator variable	Target	Effect	Boot *SE*	Boot LLCI	Boot ULCI
X1	Perspective-taking	.225	.097	.023	.533
	Female	− 064	.071	− 264	.123
	Male	.093	.065	− 059	.294
X2	Perspective-taking	.195	.088	.001	.455
	Female	− 001	.063	− 164	.180
	Male	.059	.067	− 107	.260
X3	Perspective-taking	.295	.098	.086	.579
	Female	− 035	.069	− 237	.149
	Male	.127	.068	− 025	.341

The indices of moderated mediation for fear are reported in Table 8. Again, two indices were significant, *X*2*W*1 = 0.351 (bootstrap SE = 0.137, LLCI =  − 0.758, ULCI =  − 0.038) and *X*3*W*1 =  − 0.480 (bootstrap SE = 0.153, LLCI =  − 0.923, ULCI =  − 0.132), indicating that the indirect effect of participant group on perceived sexual harassment through fear was moderated by target. The relative indirect effects at the three values of the moderator are provided in Table 9. In the perspective-taking condition, two indirect effects were significant: *X*2 = 0.374 (bootstrap SE = 0.117, LLCI = 0.109, ULCI = 0.712) and *X*3 = 0.326 (bootstrap SE = 0.116, LLCI = 0.061, ULCI = 0.673). Therefore, the relative indirect effects of the comparisons of heterosexual males with heterosexual females (*X*2) and heterosexual males with lesbians (*X*3) on perceived sexual harassment through fear were significant in the perspective-taking condition, but not in the female or male target conditions. The effect for the heterosexual males–gay males comparison (*X*1) was not significant in any target condition, partially supporting Hypothesis 3.

**Table 8:** Indices of moderated mediation (difference between conditional indirect effects) for the mediator fear

Participant group indicator variable	Target indicator variable	Index	Boot *SE*	Boot LLCI	Boot ULCI
X1	W1	− 238	.131	− 595	.100
	W2	.002	.132	− 328	.383
X2	W1	− 351	.137	− 758	− 038
	W2	− 201	.135	− 583	.135
X3	W1	− 480	.153	− 923	− 132
	W2	− 071	.136	− 430	.294

**Table 9:** Relative conditional indirect effects of participant group on perceived sexual harassment through fear

Participant group indicator variable	Target	Effect	Boot *SE*	Boot LLCI	Boot ULCI
X1	Perspective-taking	.163	.111	− 133	.461
	Female	− 075	.071	− 270	.108
	Male	.165	.079	− 022	.391
X2	Perspective-taking	.374	.117	.109	.712
	Female	.023	.070	− 167	.210
	Male	.173	.091	− 053	.432
X3	Perspective-taking	.326	.116	.061	.673
	Female	− 154	.089	− 417	.065
	Male	.255	.099	.018	.545

#### Relative Direct Effects

Although I did not predict a direct effect of participant group on perceived sexual harassment ratings, I included the direct effect in the model because this is the first proposal of this psychological process. Further, extant sexual harassment research assumes a direct effect of gender on perceived sexual harassment. The omnibus test of the relative direct effects of participant group on perceived sexual harassment was not significant (*p* > 0.01). As indicated by the effects provided in Table 10, none of the three relative direct effects were significant as all bootstrap confidence intervals contain zero. This means that, as predicted, participant group did not predict perceived sexual harassment directly but only indirectly through perceived discrimination and fear.

**Table 10:** Relative direct effects of participant group on perceived sexual harassment

Participant group indicator variable	Effect	*SE*	LLCI	ULCI
X1	− 220	.124	− 540	.100
X2	.157	.123	− 161	.474
X3	− 034	.125	− 358	.291

Thus, overall, Hypothesis 3 was largely supported. The effect of participant group on perceived sexual harassment was mediated by fear and perceived discrimination, and the effect of participant group on the two mediators was moderated by target. Heterosexual males’ ratings differed from the other participant groups in the perspective-taking condition only, except for the nonsignificant heterosexual male–gay male comparison in predicting perceived sexual harassment ratings through fear.

### Perceived Manager Gender

The manager in each scenario was gender-neutral to bolster the realism of the scenario. Participants could infer the gender of the manager based on their experiences or expectations. Toward the end of the questionnaire participants were asked to report what gender they perceived the manager to be. Table 11 reports the data for participants who responded whether they perceived the manager to be male or female. (Some participants responded that they did not know or did not respond.) Thus, Table 11 reports, for those participants that reported their perceived gender of the manager, the percentage of participants in each condition that perceived the manager to be male. Perusal of the table reveals that across conditions the manager was more likely to be perceived as male, as was the case in all but two of the twenty-four conditions.

**Table 11:** Percentage of participants perceiving manager to be male by condition

Participant group	Condition					
	Control			Sociosexual behaviors		
	PT	Mary	Mark	PT	Mary	Mark
Lesbian	78.6	63.2	83.3	58.3	31.6	87
Heterosexual female	71.4	50	72.7	71.4	62.5	62.5
Gay male	93.8	82.4	89.3	95.5	87	84.4
Heterosexual male	80	94.7	82.6	100	84	95
All participants	83%	74.6%	82.4%	82.1%	68.1%	83.5%

## Discussion

To be legally actionable, hostile work environment sexual harassment, like the unwanted sexual attention manipulated in this study, must be severe or pervasive, as determined by the court. In *Rabidue v. Osceola Refining Company* (1986), the Sixth Circuit Court of Appeals deemed that when making this assessment, the courts “must adopt the perspective of a reasonable person's reaction to a similar environment under essentially like or similar circumstances” (p. 620). The court endorsed a test that requires the plaintiff to prove both that the plaintiff was subjectively offended and injured by the impugned behaviors, and that a reasonable person would be as well. The court explained as follows:

Thus, in the absence of conduct which would interfere with that hypothetical reasonable individual's work performance and affect seriously the psychological well-being of that reasonable person under like circumstances, a plaintiff may not prevail on asserted charges of sexual harassment anchored in an alleged hostile and/or abusive work environment regardless of whether the plaintiff was actually offended by the defendant's conduct (p. 620).

The judiciary continues to be male dominated (George & Yoon, 2016). The reasonable person standard is, therefore, a de facto “reasonable man” standard. For this reason, the Ninth Circuit Court of Appeals held, in Ellison v. Brady (1991), the reasonable person standard to be inappropriate because it would “run the risk of reinforcing the prevailing level of discrimination” (p. 878). Men are not victimized at the same rate as women nor are they as vulnerable to victimization, and they do not experience sexual harassment in the same way. The court declared that a proper analysis of a hostile work environment case should adopt the perspective of the victim, giving rise to the “reasonable victim” standard. When the victim was female, the correct standard to adopt to make determinations about severity and pervasiveness is that of the “reasonable woman.”

Many courts refused to adopt the reasonable woman standard, as it came to be known (Perry et al., 2004). The standard was applied in less than 15% of the sexual harassment cases reviewed by Newman (2007); yet the standard was controversial and criticized by some scholars. For example, Blumenthal (1998) argued that the minimal gender differences produced by his meta-analysis suggested the reasonable woman standard was unnecessary. The results of this study suggest otherwise. When the participant was the target of the sociosexual behaviors, females reported higher fear and perceived sexual harassment ratings. These gender differences are consistent with findings of studies of lived experiences (e.g., Morrow et al., 1994). The same differences were not found when the target was fictional, as is often the case with labeling studies (e.g., Bursik & Gefter, 2011), which suggests that studies employing imagine-other perspective-taking may not measure participants’ own perceptions, particularly for heterosexual male participants as they likely relied on stereotypes to form inferences. This is further supported by the fact that heterosexual male participants reported higher fear, discrimination, and perceived harassment ratings when the fictional target was female than when the target was male. The stereotypical sexual harassment target is female.

Investigation of gender differences remains relevant because legislatures, the judiciary, and organizational leadership continue to be male dominated (Chappie & Humphrey, 2014; George & Yoon, 2016). Public and organizational policy regarding sexual harassment is still largely determined by men (Clarke, 2020). Men’s differential experience of sociosexual behaviors in the workplace may contribute to the insufficiency of public and organizational policy in curtailing harassment. This differential experience may prevent men, in particular heterosexual men, from truly understanding the experience of sexual harassment targets. Such perspective-taking is an antecedent to empathy and helping others (e.g., Goldstein et al., 2014). One could potentially argue that the heterosexual male participants did take the perspective of the fictional female target when they assigned higher fear, discrimination, and sexual harassment ratings to the female target condition. It is, however, more likely that the ratings resulted from labeling and stereotyping, neither of which is tantamount to understanding or empathizing. Is someone who has never experienced sociosexual behaviors as threatening or as unequal treatment able to understand why they are experienced as such by others? If not, this may help explain why sexual harassment policies and training have not sufficiently deterred harassment and why many incidents go unreported.

The results of this study also have significant implications for how scholars interpret the findings of sexual harassment studies given the study design and the use of perspective-taking. Critically important questions must be asked and examined, such as: Is this study measuring knowledge of workplace sexual harassment or experience of workplace sexual harassment? How informative are studies that group all men, or even all women, into the same category? How much is the heteronormative assumption tainting research findings?

The findings of this study aid our understanding of gender differences in the psychological process upon experiencing sociosexual behaviors in the workplace. Prior research has investigated cognitive and emotional responses as outcomes of, rather than antecedents to, perceiving behaviors to be harassment. The novel process tested and supported herein implies the psychological process that occurs after being subjected to sociosexual behaviors in the workplace may be different than previously contemplated. Although corroborating findings are necessary, it is interesting that the effect of gender on perceptions of sexual harassment was mediated by fear and perceived discrimination rather than direct. This process is consistent with the early literature on labeling, which demonstrated that targets could experience the negative outcomes of sexual harassment without labeling their experience to be harassment (e.g., Magley et al., 1999; Munson et al., 2001).

Sexual harassment is undeniably a workplace stressor; however, the findings intimate that this conceptualization is insufficient. Sexual harassment, for some, is *experienced* as discrimination. In this study, female and gay male participants, on average, perceived the sociosexual behaviors to be discrimination but the heterosexual male participants did not. Men may not find sociosexual behaviors to be as threatening as do women, but certain groups of men may experience them as unequal treatment or as putting them in their place (see Berdahl, 2007). These findings raise concerns about responses to male targets. Gay male participants reported past sexual harassment at as high a rate as the lesbian and heterosexual female participants and were equally likely to perceive the sociosexual behaviors to be discrimination. Female and gay male managers to whom incidents are reported may find it easier to understand why a male complainant feels degraded and discriminated against when the target of sociosexual behaviors in the workplace. Heterosexual male managers may perceive the male to be overly sensitive and not take the complaint seriously.

Distinguishing between gay and heterosexual males and revealing a difference between their psychological processes supplements a body of research predominated by the heterosexual norm. Further, lesbian participants did not report higher perceptions of sexual harassment or fear, but their mean discrimination rating was slightly higher than heterosexual females. A recent nationally representative study of American women found that women who were sexual minorities were more likely to report sexual harassment than heterosexual women (SteelFisher et al., 2019). A natural progression of research in this area would be to expand beyond the binary view of gender and sexual orientation and incorporate more fluid, representational measures of both variables. Future research is necessary to determine whether this study’s findings generalize to a more inclusive group of sexual minorities. For example, a recent Gallop poll found that more than half of individuals that identify as LGBT are bisexual (Schmidt, 2021). Given that the percentage of the adult population that identify as a sexual minority is predicted to continue to grow (Schmidt, 2021), the heteronormative bias pervading extant sexual harassment literature will threaten its validity.

The most significant limitation of this study is the ability of vignettes to elicit the emotional responses that would be evoked in real-life situations. However, to demonstrate the effects of study design and perspective-taking, a vignette study was necessary. Given that true experiments are ethically limited, the design is likely to be continued to be used as it is often the most effective option for testing causal relationships. The ability to test for causal relationships, along with the fact that between-subject designs are conservative tests (Charness et al., 2012), bolsters confidence in the study’s results. Rather than the relationships not generalizing to real-life situations, true effect sizes may be larger. Means and effect sizes would also likely be higher with vignettes portraying more severe or pervasive sociosexual behaviors than the single occurrence described in this study. Finally, the indirect effects found in this study should be tested in empirical studies in which participants report their perceptions of a recalled experience of workplace sexual harassment.

Another limitation is the recruitment of MTurk Workers as study participants. Although some scholars have lauded MTurk as a valuable source of experimental study participants (e.g., Landers & Behrend, 2015), studies have demonstrated that some MTurk Workers misrepresent themselves to qualify to participate in studies. That is, some MTurk workers will falsely report that they possess certain characteristics or engage in certain activities to avoid being screened out of studies (e.g., Sharpe Wessling et al., 2017). It is possible that some study participants were dishonest about their gender or sexual orientation to access the study and obtain payment.

Other researchers have argued that the concerns over MTurk are no greater than concerns over any convenience sample such as students, organizational samples, or online panels (e.g., Landers & Behrend, 2015). For example, Necka et al. (2016) compared data collected from MTurk with data collected from a sample of university students recruited on campus and a community-based sample (i.e., a sample of individuals from the Chicago general public). They examined a number of problematic behaviors that can threaten data quality (e.g., spending insufficient time reading study instructions or survey questions, socially desirable responding, falsely reporting demographics such as gender, ethnicity, or age). The results suggested that participants from all three samples engage in problematic behaviors at similar rates.

Roulin (2015) collected data from MTurk, Qualtrics, and business student samples and compared the results to data from published studies employing samples of psychology students and the general population. Roulin compared the composition of the samples as well as their scores on a measure of competitive worldviews. MTurk Worker scores on the competitive worldviews measure were similar to the general population and had reliability coefficients that were “excellent and equivalent” to the general population. MTurk scores also had the least range restriction of the convenience samples and the MTurk samples were more diverse. Similarly, Casler et al. (2013) found their sample of MTurk workers to be more socioeconomically and ethnically diverse than their samples of individuals recruited through social media and undergraduate students. MTurk was likely the best choice for recruitment in this study as it is more representative of the general population than other convenience samples and permitted the recruitment of equal numbers by gender and sexual orientation.

Despite legal prohibitions, organizational interventions, and the voluminous body of knowledge that has accumulated on workplace sexual harassment, it continues to permeate workplaces, corrupt relationships, and damage the lives of many. Over one-third of the gay male, lesbian, and heterosexual female participants in this study had been targets of sexual harassment. The findings of this study indicate that further research is needed to better understand this construct and that the stressor–strain model may be an insufficient framework to explain the psychological experience of sexual harassment. The differential experience of sociosexual behaviors may help explain why sexual harassment continues to plague contemporary workplaces, why women continue to be targeted at higher rates, why men continue to perpetrate harassment at higher rates, and why public and organizational policies have failed to have a significant impact on the phenomenon. This understanding is crucial to promoting the respect, dignity, and well-being of all employees and to addressing the legal, business, and ethical implications of sexual harassment for employers.

## Data Availability

All data and study materials are available from the author.

## Perspective-taking (Participant as Target) Scenarios

### Both Conditions

Imagine that you are employed with International Widget Manufacturers Inc., a company that manufactures widgets. Five years ago, you were hired as a Sales Representative. The manager to whom you directly report is the Regional Sales Manager. The job is enjoyable and meaningful to you. To date, you have received good performance appraisals and feedback from your manager, colleagues, and customers, but lately you have felt bored with your job. Recently, a position opened up in the Finance Department. You decide to apply for the position so that you can try something new.

## Control Condition

One day, you are eating in the lunchroom at work when your manager enters the room. You say hello and your manager smiles and says “I hear that you applied for the finance/HR position.” You reply “Yes I have.” Your manager then asks you some general questions about where you worked before you came to International Widgets and wishes you luck getting the job.

## Sociosexual Behavior Condition

One day, you are eating in the lunchroom at work when your manager enters the room. You say hello and your manager smiles and looks you up and down, sizing you up. Your manager crosses the room and stands so close to you that your bodies touch. You try to start a conversion but before long your manager turns the focus of the conversation from general “small talk” to asking you personal questions regarding your sex life.

## Female Target Scenarios

### Both Conditions

Mary has been employed with International Widget Manufacturers Inc., a company that manufactures widgets. Five years ago, Mary was hired as a Sales Representative. The manager to whom she directly reports is the Regional Sales Manager. The job is enjoyable and meaningful to her. To date, she has received good performance appraisals and positive feedback from her manager, colleagues, and customers, but lately she has felt bored with her job. Recently a position opened up in the Finance Department. Mary decides to apply for the position so that she can try something new.

## Control Condition

One day, Mary is eating in the lunchroom at work when her manager enters the room. Mary says hello and her manager smiles and says “I hear that you applied for the finance position.” Mary replies “Yes I have.” Her manager then asks her some general questions about where she worked before she came to International Widgets and wishes Mary luck getting the job.

## Sociosexual Behavior Condition

One day, Mary is eating in the lunchroom at work when her manager enters the room. Mary says hello and her manager smiles and looks her up and down, sizing her up. Her manager crosses the room and stands so close to her that their bodies touch. Mary tries to start a conversion but before long her manager turns the focus of the conversation from general “small talk” to asking her personal questions regarding her sex life.

## Male Target Scenarios

Identical to the female target scenarios with “Mark” substituted for “Mary” and feminine pronouns replaced with masculine pronouns.
